# CMV hyperimmune globulin as salvage therapy for recurrent or refractory CMV infection in children undergoing hematopoietic stem cell transplantation

**DOI:** 10.3389/fped.2023.1197828

**Published:** 2023-07-24

**Authors:** Melissa Panesso, María Luz Uría, Berta Renedo, Juliana Esperalba, María Isabel Benítez-Carabante, Natalia Mendoza-Palomar, Laura Alonso, Maria Oliveras, Cristina Diaz-de-Heredia

**Affiliations:** ^1^ Division of Pediatric Oncology and Haematology, Hospital Universitari Vall d’Hebron, Barcelona, Spain; ^2^ Institut de Recerca Vall d’Hebron, Barcelona, Spain; ^3^ Pharmacy Division, Hospital Universitari Vall d’Hebron, Barcelona, Spain; ^4^ Microbiology Division, Hospital Universitari Vall d’Hebron, Barcelona, Spain; ^5^ Pediatric Infectious Diseases and Immunodeficiencies Unit, Hospital Universitari Vall d’Hebron, Universitat Autònoma de Barcelona, Barcelona, Spain

**Keywords:** cytomegalovirus, hematopoietic stem cell transplantation, children, CMV hyperimmune globulin, salvage therapy

## Abstract

Cytomegalovirus (CMV) is a major cause of allogeneic hematopoietic stem cell transplant (HSCT)-related morbidity and mortality. Treatment failure continues to be a major issue in patients with CMV infection due to both drug resistance and intolerance. This single-center brief retrospective analysis of a case series aims to investigate the safety and efficacy of CMV-hyperimmune globulin as salvage therapy for CMV infection in children undergoing HSCT. Fifteen pediatric patients received human CMV-specific immunoglobulin (CMVIG) between July 2018 and December 2021 as a salvage therapy for refractory or recurrent CMV infection. At the time of CMVIG prescription, eight children presented with recurrent CMV infection and seven with refractory CMV infection. The overall response rate was 67% at 50 days from the CMVIG administration [95% confidence interval (CI): 44–88]. Overall survival (OS) from CMVIG administration at 100 days was 87% (95% CI: 56–96), and OS from HSCT at 1 year was 80% (95% CI: 50–93). Four patients died, three unrelated to CMV infection and one due to CMV pneumonia. CMVIG as salvage therapy was well tolerated, and no infusion-related adverse events were observed.

## 1. Introduction

Cytomegalovirus (CMV) is the most common cause of viral infection after allogeneic hematopoietic stem cell transplant (HSCT) (1). Humans suffer a CMV primary clinical or subclinical infection during infancy or childhood; CMV then remains in a latent state in several types of leucocytes and hematopoietic progenitors under the control of T cells (2). The severe and prolonged lymphocytopenia and T-cell dysfunction associated with HSCT may cause CMV reactivation, systemic viral infection, and ultimately CMV organ diseases such as pneumonia, colitis, and others. The main risk factors for CMV disease are the recipient CMV-positive serology, using donors other than matched sibling donors, the cord blood as a stem cell source, *in vivo* or *ex vivo* T-cell depletion, the occurrence of graft-vs.-host disease (GVHD), and using steroids and other immunosuppressants to treat GVHD (3). CMV disease can be a direct cause of mortality, but also CMV infection can be an indirect cause of lower overall survival (OS) and non-relapse mortality in allogeneic HSCT recipients (4). The main strategies for CMV prevention include donor selection, careful use of immunosuppressants, antiviral prophylaxis, and early treatment of CMV reactivation (preemptive approach). Despite the efficacy of ganciclovir prophylaxis, its myelotoxicity precludes its use in the HSCT setting. Although the safer alternative letermovir has been licensed for CMV prophylaxis in adults, it has not yet been authorized for children. Thus, preemptive therapy remains the mainstay to prevent CMV disease in pediatric HSCT recipients. This approach requires regular blood screening for detecting active CMV replication using adequate techniques to start an antiviral treatment upon detecting significant viremia or antigenemia (5). However, in patients with CMV infection, CMV treatment failure continues to be a major issue (6). The currently available antivirals have high toxicity, might produce bone marrow suppression, and are not always efficient in clearing the virus. Cytopenia, which can greatly increase the risk of bacterial and fungal coinfections, is the most important side effect of ganciclovir/valganciclovir therapy, while renal impairment is the main side effect of foscarnet (7). Cidofovir has activity against some ganciclovir-resistant CMV isolates. Therefore, it could be used as a third-line treatment in patients with refractory CMV infection. Nevertheless, like foscarnet, cidofovir is responsible for renal impairment (8). There are limited data on the efficacy and toxicities associated with CMV antiviral regimens utilized in pediatric allogeneic HSCT recipients, so treating CMV infection represents a great challenge in this population (7).

CMV-specific immunoglobulin (CMVIG) is a hyperimmune globulin obtained from plasma donors with high titers of CMV-specific antibodies (100 U of CMV-specific antibodies per milliliter). Previous studies have compared titers of CMV-specific antibodies in CMVIG and standard intravenous immunoglobulin (IVIG) preparations. Data presented by Miescher et al. (9) and Gupta et al. (10) demonstrate the higher anti-CMV neutralization capacity of CMVIG per gram of IgG vs. standard IVIG. These findings suggest that standard IVIGs are not equivalent to or interchangeable with CMVIG at the same doses. CMVIG binds to the antigens on the surface of CMV and, through this mechanism, neutralizes the capacity of CMV to enter the host cells. It also exhibits complex immunomodulatory actions that might contribute to controlling the effects of the virus infection (11). CMVIG (Megalotect®) is authorized for use in patients receiving immunosuppressive treatment to prevent the clinical manifestation of cytomegalovirus infection, particularly in patients after organ transplantation. In an adult setting, CMVIG was used as preemptive/rescue therapy in allogeneic HSCT with promising results. However, there is a scarce experience in the pediatric setting for this indication (12). Herein, we present our center’s experience of using CMVIG as a salvage therapy for CMV infection in pediatric patients undergoing HSCT. The primary goal of this study was to evaluate CMVIG’s safety and efficacy in pediatric patients.

## 2. Methods

This single-center retrospective analysis was conducted at the Pediatric Hematology and Oncology Division, Hospital Vall d'Hebron, Barcelona, Spain. Datasets from children who underwent allogeneic HSCT and received CMVIG between 2018 and 2021 were analyzed retrospectively.

The study data were obtained from the transplant unit databases and the patients' medical records. Ethical standards and legal requirements on the use of personal data were applied throughout the data collection stages. The use of CMVIG for refractory/recurrent CMV infection was approved by the institutional review board. Parents or legal guardians of the patients gave their informed consent for off-label CMVIG use.

### 2.1. Inclusion criteria

Children <18 years old who underwent allogeneic HSCT with refractory or recurrent CMV infection unresponsive to antiviral drug therapy and received CMVIG as salvage therapy were included. Patients must not have received escalation of the antiviral treatment in the 2 weeks prior to the start of the CMVIG administration.

### 2.2. Endpoints

The primary endpoint was the overall response rate (ORR). Secondary endpoints were CMV subsequent reactivation after discontinuation of CMVIG, OS after the administration of CMVIG, and OS after allo-HSCT.

### 2.3. Quantification of CMV DNAemia

Posttransplant CMV DNAemia was quantified once or twice weekly in whole blood by qPCR using the Altona manufacturer assay. DNAemia ≥1,000 IU/mL (log^10^ IU/mL ≥3) was used to initiate antiviral drug treatment. In all patients the viral load was quantified within 3 days of the start of CMVIG therapy.

### 2.4. Definitions

*Refractory CMV infection* was defined as the absence of a decline in CMV DNAemia of at least 0.5 log^10^ IU/mL despite administering full-dose antiviral drug therapy for >2 weeks.

*Recurrent CMV infection* was defined as the new detection of CMV infection in a patient who had previously presented CMV DNAemia, but it remained undetectable for at least 4 weeks after antiviral drug therapy.

*Response* was defined as two negative results in consecutive weeks of CMV DNAemia since the initiation of CMVIG without changing antiviral treatment or adding a new CMV therapy.

*Time to response* was the time from the start of CMVIG until a response was obtained.

*Subsequent reactivation* was defined as positive CMV DNAemia after discontinuation of CMVIG, having achieved a response.

*CMV disease* was defined as the presence of signs and symptoms of organ damage along with the detection of infection by directly identifying the virus or any of its components with various techniques (histology or detection of DNA).

### 2.5. CMV-resistant strains testing

The study of CMV genetic mutations that are associated with resistance to antivirals ganciclovir, foscarnet, and cidofovir was carried out by means of Sanger sequencing of the UL54 (polymerase) and UL97 (kinase) regions. Sequence editing was performed by the MEGA v5.0 program using the consensus sequences for each region, and mutation analysis was performed using the public MRA-mutation resistance analyzer database of the University of Ulm (13).

### 2.6. CMVIG dosage

CMV hyperimmune globulin dosage was given according to the posology used by Alsuliman et al.: 400 U/kg on days 1, 4, and 8 and then 200 U/kg on days 12 and 16 (12).

### 2.7. Patient follow up

Patients were followed up after transplant to monitor whether they experienced CMV reactivation and after CMVIG administration to monitor infusion-related adverse effects, response to treatment, and subsequent reactivations. The follow-up of the patients was carried out until the last visit prior to the end date of the study or until their death.

### 2.8. Statistical analysis

Categorical variables were described as frequencies, and continuous variables were described as median values. The time to response was calculated in days. The probability of overall response was estimated using the Kaplan–Meyer estimator as the interval from the first CMVIG infusion to response, and patients were examined at the date of death or the date of last contact if alive. The LogRank test was used to compare patients with recurrent CMV infection and those with refractory CMV infection. The time to follow-up was calculated in months (median and range), and OS and cause of death were collected from the date of transplant and from the date of the first CMVIG infusion. The probabilities of OS were estimated using the Kaplan–Meyer estimator as the interval from CMVIG administration to death and the interval from allo-HSCT to death, whatever the cause, and patients were examined at the date of the last contact if alive.

## 3. Results

**Figure 1:**
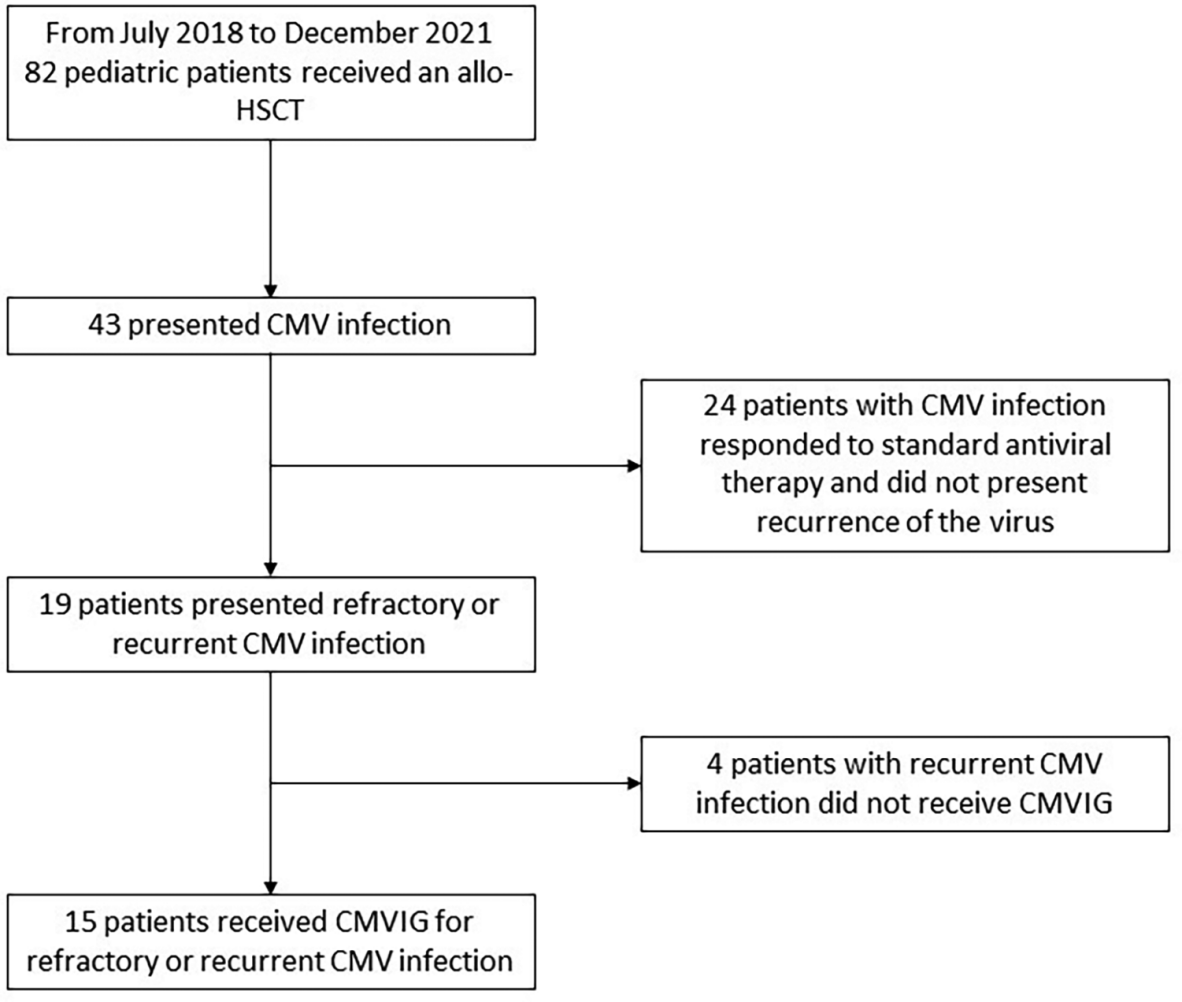
Enrollment and treatment.

From July 2018 to December 2021, the Pediatric Hematology and Oncology Division of the Hospital Universitari Vall d’Hebron, Spain, performed allogenic HSCT in 82 pediatric patients. From those, 15 pediatric patients (7 boys and 8 girls) presented refractory or recurrent CMV infection and consented to receive CMVIG as rescue therapy (Figure 1). The patients’ and transplants’ characteristics are described in Table 1. All patients had received posttransplant antiviral prophylaxis with acyclovir. Recurrent/refractory CMV infection appeared mostly in patients who had undergone unrelated or mismatch transplantation (93.3%), with *in vivo* T-cell depletion (93.3%) and a serologic negative CMV donor for a positive serologic recipient (33%), very poor immune reconstitution, and suffering acute GVHD II–IV (53%), receiving steroids in a dosage ≥0.5 mg/kg/day (67%), and having other viral infections (73%).

**Table 1:** Patient and transplant characteristics.

Patient	Age (years)	Underlying disease	Previous HSCT	Donor	Source of stem cells	Receptor/Donor CMV serostatus	Conditioning regimen	*In vivo* T-cell depletion	*Ex vivo* T-cell depletion	aGVHD (grade)	Steroids >0.5 mg/kg/day	Lymphocyte count ×10E9/L (total/CD4) at CMVIG prescription	Days from HSCT to first CMV reactivation	Other viral infections
1	≥5–10	Amegakaryocytic thrombocytopenia	Yes	Haploidentical	BM	R+/D+	RIC	ATG	No	0	No	0/0	19	AdV, EBV, HHV-6
2	≥10–18	Lymphoblastic lymphoma T, CR-2	Yes	Haploidentical	PB	R+/D+	RIC	ATG	Yes	2	Yes	0.8/0.1	14	AdV, EBV
3	0–5	PID (mut. ARPC1b)	No	MMUD	BM	R+/D−	RIC	ATG	No	0	No	0/0	8	No
4	≥10–18	ALL-B, CR-2	No	MMUD	BM	R+/D−	MAC	ATG	No	1	No	0.2/0.01	26	AdV, EBV
5	≥10–18	ALL-B, CR-1	No	MMUD	BM	R+/D+	MAC	ATG	No	1	Yes	0.2/0.05	25	EBV
6	≥10–18	Fanconi anemia	No	MUD	PB	R+/D+	RIC	ATG	Yes	0	No	0.7/0.01	20	No
7	≥10–18	ALL-T, CR-2	No	MUD	PB	R+/D−	MAC	ATG	No	2	Yes	0.5.0.04	29	AdV, EBV, HHV-6
8	0–5	Thalassemia maior	No	MSD	CB	R+/D+	MAC	No	No	2	Yes	0.7/0.05	25	No
9	≥10–18	ALL-B, CR-1	No	MMUD	PB	R+/D+	MAC	ATG	No	4	Yes	0.3/0.04	24	AdV, EBV
10	0–5	Chronic granulomatous disease	No	MUD	BM	R+/D+	RIC	Alemtuzumab	No	2	Yes	0.1/0.01	30	EBV, BK
11	≥10–18	ALL-B, CR-1	No	MUD	BM	R+/D+	MAC	ATG	No	1	Yes	2.4/0.5	50	AdV, EBV
12	≥5–10	ALL-B, CR-2	No	MUD	BM	R+/D−	MAC	ATG	No	1	No	0.05/0.05	21	No
13	≥10–18	Chronic mucocutaneous candidiasis	No	MMUD	PB	R+/D+	RIC	ATG	No	2	Yes	0/0	1	BK, HHV-6, Parvovirus B-19
14	≥10–18	Chronic granulomatous disease	No	MUD	BM	R+/D−	RIC	Alemtuzumab	No	2	Yes	0.2/0.04	18	BK
15	≥10–18	ALL-B, CR-1	No	MMUD	PB	R+/D+	MAC	ATG	No	2	Yes	0.2/0.02	25	EBV, BK

F, female; M, male; MMUD, mismatched unrelated donor; MUD, matched unrelated donor; MSD, matched sibling donor; BM, bone marrow; PB, peripheral blood; CB, cord blood; RIC, reduced intensity conditioning; MAC, myeloablative conditioning; ATG, anti-thymocyte globulin; aGVHD, acute graft-vs.-host disease; EBV, Epstein-Barr virus; BK, BK virus; AdV, adenovirus; HHV-6, human herpesvirus 6.

The median time from HSCT to the first CMV reactivation was 25 days (range: 1–50). First-line antiviral treatment was started in all patients with ganciclovir/valganciclovir or foscarnet according to the toxicity profile. In some of them, second-line treatment was started because of refractory CMV infection or to treat coinfection with other viruses, but no escalation of the antiviral treatment was made in the 2 weeks prior to the start of the CMVIG.

Seven patients received CMVIG for refractory CMV infection, eight for recurrent infections (three in the second reactivation episode, four in the third, and one in the fourth). Of the patients with recurrent CMV infection, two were on active antiviral treatment at the moment of the reactivation episode for which they received the CMVIG therapy, and four stopped the treatment within 21 days of reactivation. Resistance testing was done in five patients, with negative results. The median viral load at the time of CMVIG administration was 3.80 log^10^ IU/mL (range: 2.76–5.12). All patients received CMVIG in combination with antiviral drugs (ganciclovir/valganciclovir in five, foscarnet in five, ganciclovir + foscarnet in one, and cidofovir in four).

The administration of CMVIG was very well tolerated in this case series; no infusion-related adverse events (AEs) were detected.

**Figure 2:**
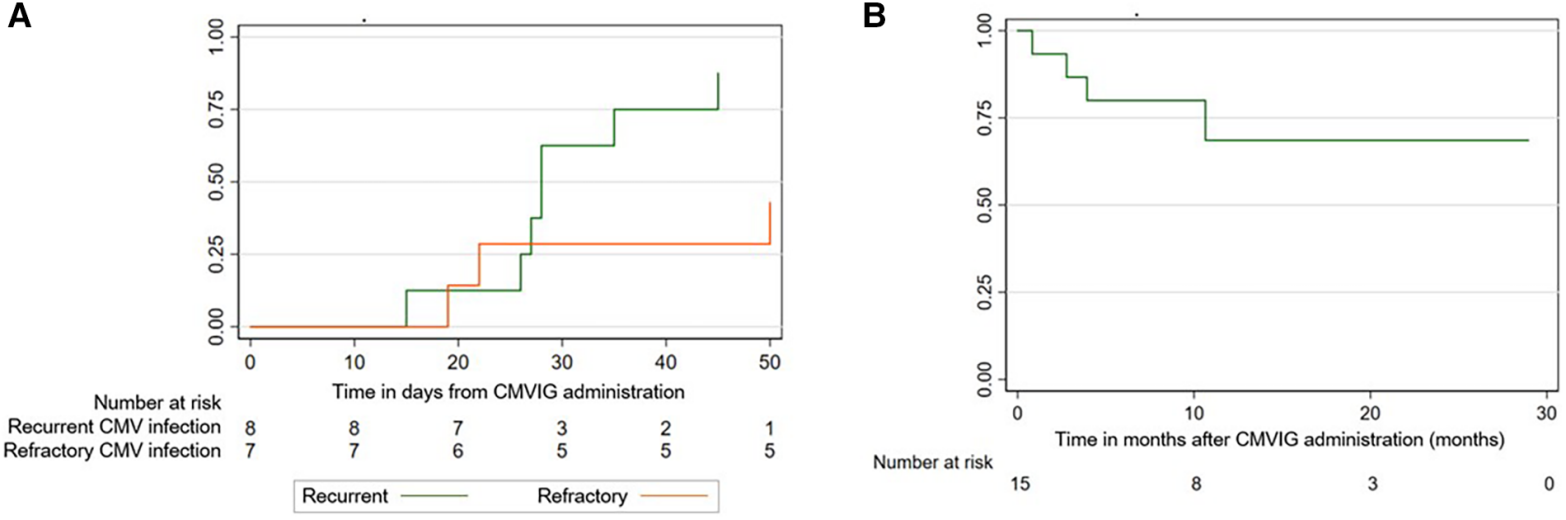
(**A**) Response rate: recurrent vs. refractory CMV infection. (**B**) Overall survival after CMVIG administration.

Supplementary Figure shows the evolution of the viral load after the start of CMVIG therapy. The ORR was 67% at 50 days from CMVIG administration [95% confidence interval (CI): 44–88]. The response rate was higher in patients with recurrent CMV infection [87% at 50 days (95% CI: 58–99)] compared to patients with refractory CMV infection [43% at 50 days (95% CI: 16–83)], although this result was not statistically significant (Figure 2A). The median time to achieve a response was 27 days (range: 15–50). Four patients with recurrent CMV reactivation and response to the administration of CMVIG (50%) suffered subsequent reactivation, with the days to subsequent reactivation ranging from 15 to 60. Three CMVIG non-responder patients received CMV-specific cytotoxic T-lymphocytes (CTLs).

The median post-CMVIG administration follow-up period was 10 months (range: 1–29). OS from CMVIG administration at 100 days was 87% [95% CI: 56–96] (Figure 2B). The median posttransplant follow-up period was 12 months (range: 3–38). OS from HSCT was 80% at 1 year [95% CI: 50–93]. Four patients died at 3-, 7-, 7- and 13-month post-transplantation. Two of these patients were CMVIG responders (20% of responders), and two were CMVIG non-responders (40% of non-responders). Three deaths were unrelated to CMV infection, while one patient died from CMV pneumonia. Each patient's treatment characteristics and outcomes are summarized in Table 2.

**Table 2:** Patient's treatment characteristics and outcomes.

Patient	Episode of CMV when CMVIG was used	Recurrent vs refractory CMV infection	CMV Viral load (log)	Days from HSCT/CMV infection to CMVIG	Antiviral tratment (with CMVIG)	Response/Days to response	Subsequent reactivation/days to subsequent reactivation	CMV-specific cytotoxic T-lymphocytes	Post-HSCT follow-up (months)	Current status/Cause of death
1	1	Refractory	4.00	50/31	Cidofovir	No	Not applicable	No	3	Died/acute hemorrhage
2	2	Recurrent	3.99	73/59	Cidofovir	Yes/45	No	No	7	Died/leukoencephalopathy
3	1	Refractory	3.38	9/0	Foscarnet	No	No	No	27	Alive
4	3	Recurrent	4.55	126/100	Ganciclovir	Yes/16	Yes/30	No	30	Alive
5	2	Recurrent	3.45	270/245	Foscarnet	Yes/28	Yes/26	No	38	Alive
6	1	Refractory	4.75	48/28	Valganciclovir	No	Not applicable	Yes	13	Died/CMV pneumonia
7	2	Recurrent	4.60	231/202	Cidofovir	Yes/15	No	No	24	Alive
8	1	Refractory	5.09	55/30	Ganciclovir + Foscarnet	Yes/22	No	No	12	Alive
9	3	Recurrent	3.24	125/99	Cidofovir	Yes/35	Yes/15	No	7	Died/invasive fungal infection
10	4	Recurrent	3.80	217/187	Foscarnet	Yes/28	Yes/60	No	18	Alive
11	1	Refractory	5.12	73/23	Ganciclovir	Yes/19	No	No	12	Alive
12	3	Recurrent	3.43	155/134	Ganciclovir	No	Not applicable	Yes	14	Alive
13	1	Refractory	2.76	62/61	Foscarnet	No	Not applicable	Yes	8	Alive
14	3	Recurrent	3.64	179/161	Ganciclovir	Yes/27	No	No	16	Alive
15	1	Refractory	3.36	63/38	Foscarnet	Yes/50	No	No	8	Alive

## 4. Discussion

CMV infection can cause morbidity and mortality in the setting of immunodeficiency, including the immune reconstitution phase following allogeneic HSCT (14). In the last three decades, the mortality rate from CMV disease has decreased significantly with the preemptive therapy approach. Nevertheless, the percentage of patients who at some point present infection or reactivation of the virus after allogeneic HSCT is high. This seems especially relevant with the rise of alternative forms of allo-HSCT (mismatched, haploidentical, *ex vivo* T-cell depletion), all of which increase the risk of viral infections. Given that most of these patients receive specific treatment and that the antiviral agents currently used as first-line treatment have significant adverse effects and toxicity, secondary morbidity continues to be a significant complication of preemptive treatment. Furthermore, treatment failure continues to be a major issue in patients with CMV infection due to both drug resistance and intolerance. In our series, despite having carried out a resistance study in five of the patients, infection by CMV-resistant strains could not be confirmed. In fact, drug resistance seems uncommon in CMV infections after allogeneic HSCT, but it can still occur during CMV prophylaxis or treatment and should be suspected in patients who increase their viral load for more than 2 weeks despite well-conducted therapy. New promising treatments for refractory CMV infections with or without drug resistance, like Maribavir, are not yet authorized for children (15).

Being aware that cellular and humoral mechanisms are involved in the immune response to CMV infection, of the drawbacks related to the use of current antivirals, and of the possibility of drug resistance, there is an obvious need for other options to prevent and treat CMV in pediatric allogeneic HSCT patients (16).

Given its good safety profile, some groups considered CMVIG as an additional option for prophylaxis in pediatric HSCT. Recent data on CMVIG prophylaxis in pediatric HSCT were reported by Geurten et al. in a single-center retrospective study comparing the incidence and severity of CMV infection with and without prophylactic CMVIG. Although the sample size was insufficient to generate conclusions, a slight reduction in the incidence of CMV infection was observed without any severe or refractory infection in the group of patients receiving prophylactic CMVIG (17).

Despite not currently being used as a first-line treatment in allogeneic HSCT patients, CMVIG is a therapeutic option to be considered in this context and in addition to antiviral treatment. CMVIG demonstrated a good safety and efficacy profile in an adult allogeneic HSCT setting (12), but the experience with CMVIG as a rescue therapy for refractory/recurrent CMV infections in allogeneic HSCT in children has been rarely reported. Our major finding in this study is that CMVIG in children could be an option as a salvage therapy, with an ORR of 67% at 50 days [95% CI: 44–88] and an OS of 87% [95% CI: 56–96] at 100 days from the CMVIG administration. The absence of infusion-related AEs in our study supports using CMVIG as a safe treatment option. These results are in line with those reported by Alsuliman et al. in an adult setting. These findings might suggest that CMVIG, in addition to antiviral treatment, could help in the control of CMV infection with the advantage of good tolerance.

In another retrospective study conducted by Malagola et al. (18), 78 patients, 6 of whom were pediatric patients, received CMVIG in first-line preemptive therapy in addition to conventional antiviral agents. After a median of 20 days of therapy, 51 out of the 78 patients (65%) achieved complete clearance of CMV viremia. However, the characteristics and results of the pediatric population are not specifically described. In addition, unlike our study, the CMVIG therapy was not used in the context of recurrent/refractory CMV infection, and the CMVIG dosage was variable, making it difficult to compare the results between both studies.

According to the data presented in our study, the presence of refractory or recurrent CMV infection could represent an indication for treatment with CMVIG. In the case of refractory infections, after carrying out the resistance study when suspected and contemplating combination therapy or changing the antiviral agent, CMVIG would be indicated. In the context of intolerance to antiviral treatments, CMVIG could also be considered a therapeutic option.

In recurrent infections, the scenario is more heterogeneous, and the decision to use CMVIG in a specific episode seems to be determined by several factors, such as the reactivation episode and the time elapsed since the previous one, the presence of active treatment for the virus or secondary prophylaxis, time since HSCT, immune reconstitution status, and the assessment of risk factors for CMV reactivation or disease. After successful use of CMVIG in recurrent CMV, in subsequent reactivation episodes, administration of a new course of CMVIG could be considered in addition to other alternatives apart from adjusting antiviral treatment according to the patient's immune status.

In this study, three patients received CTLs in the context of a lack of response to the administration of CMVIG and optimization of the antiviral treatment. One of them finally died from CMV pneumonia; the other two patients had a satisfactory evolution with complete remission of the CMV infection to the follow-up date. CTLs have a vital role in the control of CMV infection or disease, and adoptive immunotherapy with CMV-CTLs, obtained from their CMV-seropositive stem cell donors or CMV-seropositive third-party donors, has been used as a treatment of CMV infection in allogeneic HSCT recipients (19–21); some studies have shown treatment benefits with a good safety profile and feasibility (18, 22).

This study has several limitations. The most important to mention are the low number of patients included, being a single-center cohort, the lack of a control group and risk stratification, the difficulties of evaluating the efficacy in patients receiving multiple treatments simultaneously, and its retrospective nature. In addition, despite the fact that CMVIG was administered in this group of patients according to the posology used by Alsuliman et al., there are no established protocols for administering CMVIG in this indication.

In conclusion, CMVIG as a salvage therapy seemed effective and safe in children with recurrent or refractory CMV infection after HSCT. A large prospective study is needed to confirm these results.

## Data Availability

The original contributions presented in the study are included in the article/**Supplementary Material**, further inquiries can be directed to the corresponding author.
