# Supplementary material for: CMV hyperimmune globulin as salvage therapy for recurrent or refractory CMV infection in children undergoing hematopoietic stem cell transplantation
Source: Front Pediatr. 2023 Jul 24;11:1197828. doi: 10.3389/fped.2023.1197828 (PMC10405925; doi:10.3389/fped.2023.1197828)
Supplement: Supplementary file 1 [file Presentation1.pptx]

## Slide 1
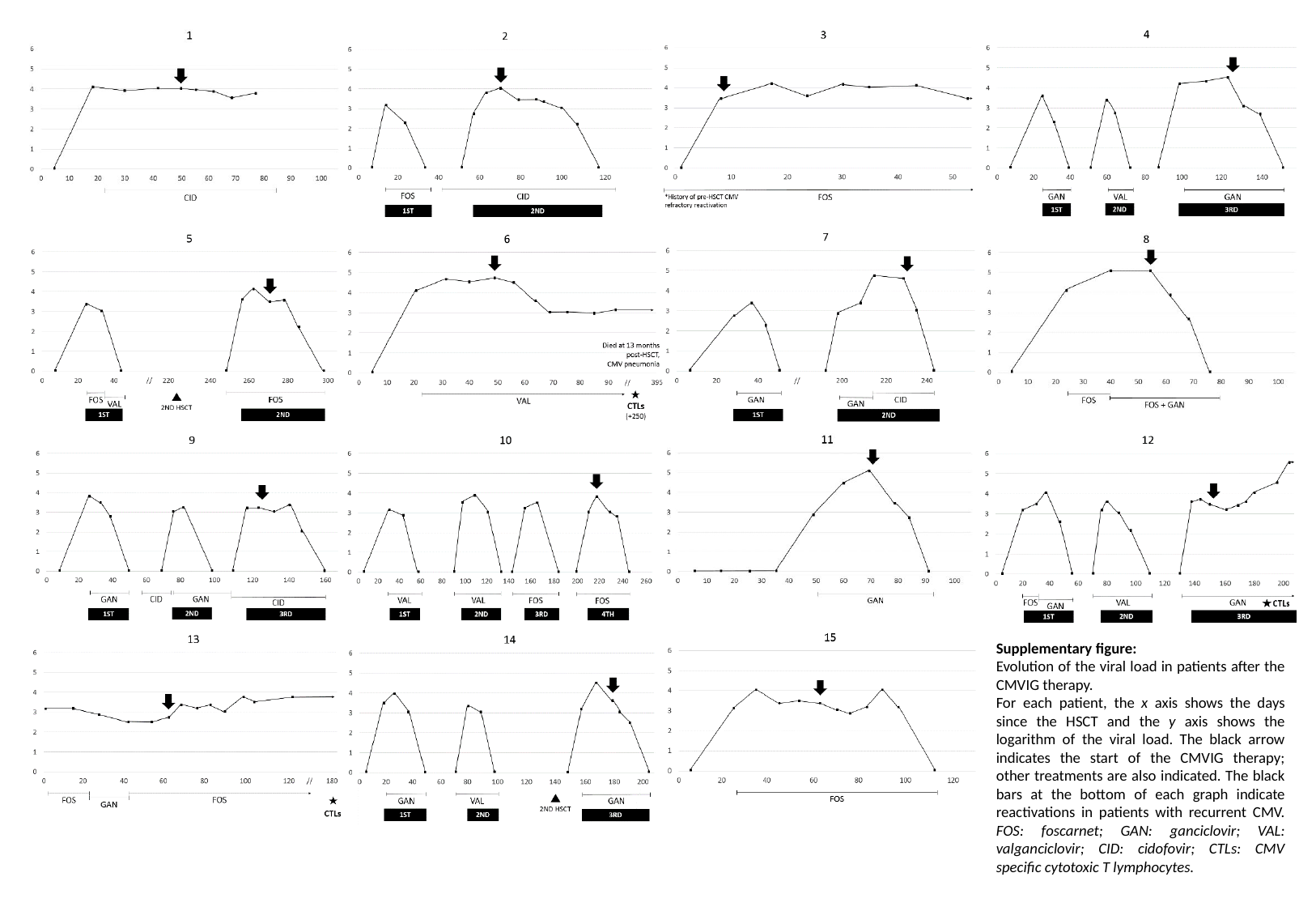

Supplementary figure:
Evolution of the viral load in patients after the CMVIG therapy.
For each patient, the x axis shows the days since the HSCT and the y axis shows the logarithm of the viral load. The black arrow indicates the start of the CMVIG therapy; other treatments are also indicated. The black bars at the bottom of each graph indicate reactivations in patients with recurrent CMV. FOS: foscarnet; GAN: ganciclovir; VAL: valganciclovir; CID: cidofovir; CTLs: CMV specific cytotoxic T lymphocytes.
